# Giant Aortic Arch Aneurysm and Cardio-vocal Syndrome: Still An Open-surgery Indication

**DOI:** 10.4021/cr101w

**Published:** 2011-11-20

**Authors:** Jose M. Garrido, Maria Esteban, Juan Lara, Jose F. Rodriguez-Vazquez, Samuel Verdugo-Lopez, Salvador Lopez-Checa

**Affiliations:** a Department of Cardiac Surgery, Department of Anatomy and Embryology II, Ramon y Cajal Hospital, Complutense University, Madrid, Spain; b Department of Cardiovascular Surgery, Virgen de las Nieves Hospital, Granada, Spain; c Department of Anatomy and Embryology II, Complutense Unversity, Mardid, Spain

**Keywords:** Aortic aneurysm, Cardio-vocal syndrome, Ortner’s Syndrome

## Abstract

The Cardio-vocal Syndrome (Ortner’s syndrome) is described as hoarseness due to the left recurrent laryngeal nerve palsy, caused by a specific cardiovascular pathology. In this case, we present a patient with a giant aortic arch aneurysm with an initial clinical presentation of Cardio-vocal Syndrome. The conventional open-surgery, instead of endovascular approach, was useful to control the morbidity from the compressive effect of adjacent structures, also preventing the aortic rupture. We strongly recommend analyzing carefully the individual case and the clinical targets to resolve, because the new technologies are not always the most effective therapeutic response.

## Introduction

The Cardio-vocal syndrome (Ortner’s syndrome) [1] is produced by the palsy of the left recurrent laryngeal nerve due to different cardiovascular diseases. It was initially associated with left atrium enlargement and mitral valve pathology. In this sense, vocal cord paralysis is caused by a compression of the left recurrent laryngeal nerve between the pulmonary artery (enlarge and hypertensive in end-stage mitral valve disease), the aorta, and the ligamentum arteriosum. However other causes have been recently described such as penetrating atherosclerotic ulcers of the thoracic aorta [2], atrial mixoma, mitral regurgitation, congenital heart disease or, rarely, aortic aneurysms.

In this work, we present a patient with a giant aortic arch aneurysm diagnosed from a cardio-vocal syndrome presentation. A saccular aneurysm of the aortic arch is a very rare cause of hoarseness. This symptom, isolated or together with the presence of dysphagia, dyspnea or chest pain, constitutes a clear indication for surgical treatment of patients with aortic arch aneurysms [3].

## Case Report

We present a 75 years old man, ex-smoker, with a history of arterial hypertension, chronic obstructive pulmonary disease and chronic renal failure. Since the past month, the patient has clinical symptoms of hoarseness and progressive dysphagia. This situation has advanced to dyspnea and thoracic pain in the last week.

**Figure 1:**
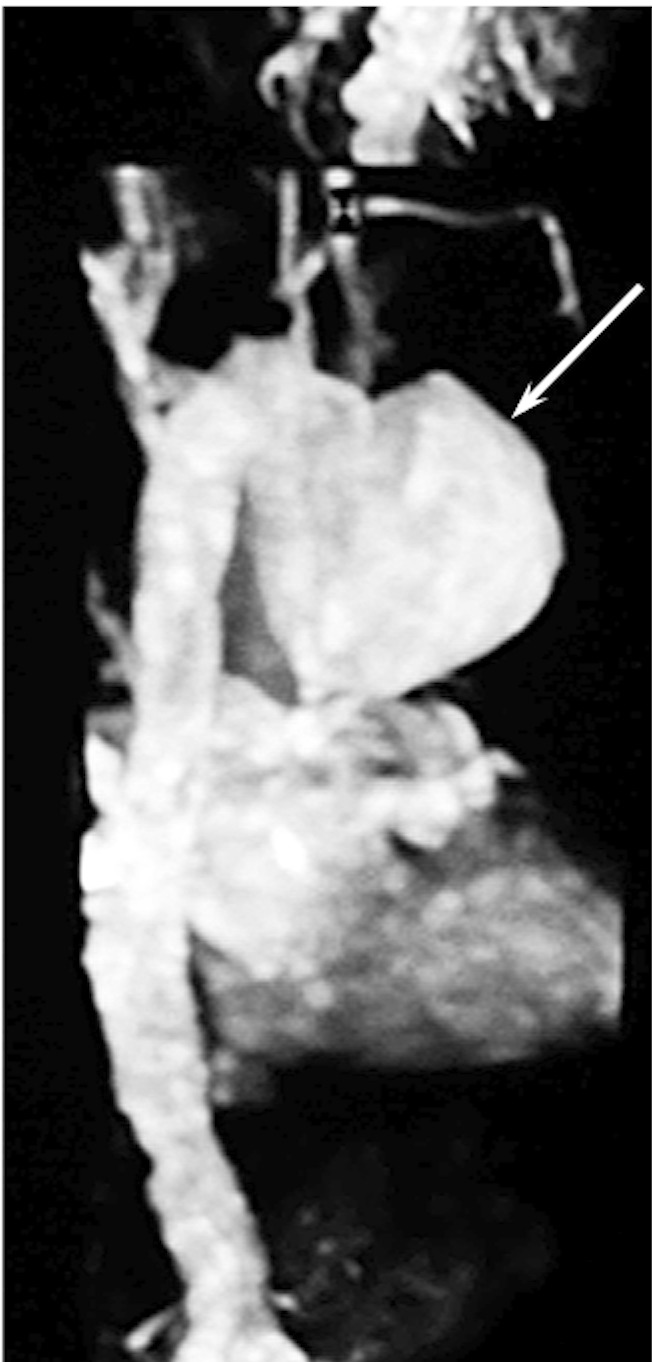
Giant saccular aortic arch aneurysm (white arrow) diagnosed by magnetic resonance imaging (MRI).

**Figure 2:**
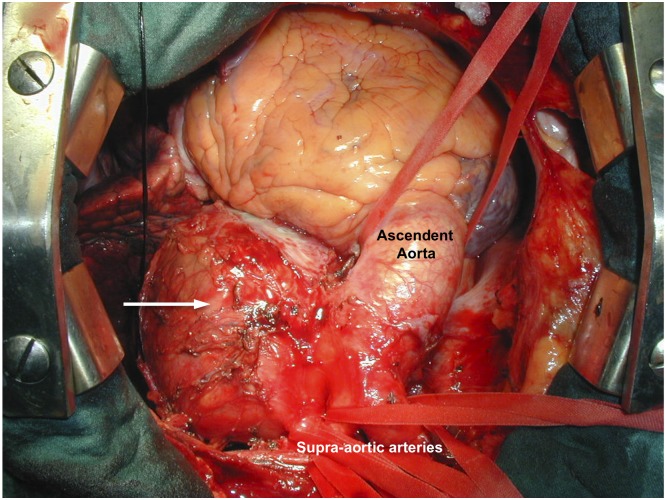
Intra-operative view of a giant aortic arch aneurysm (white arrow).

Using magnetic resonance imaging (Fig. 1), we diagnosed a giant saccular aneurysm involving the basal portion of the aortic arch, at the opposite side of the origin of supra-aortic arteries, with a significant mural thrombus, compressing the trachea, the left recurrent laryngeal nerve at the aortopulmonary window and the oesophagus [4]. The dimensions were 15 cm of external diameter and 8 cm of internal diameter (Fig. 2).

**Figure 3:**
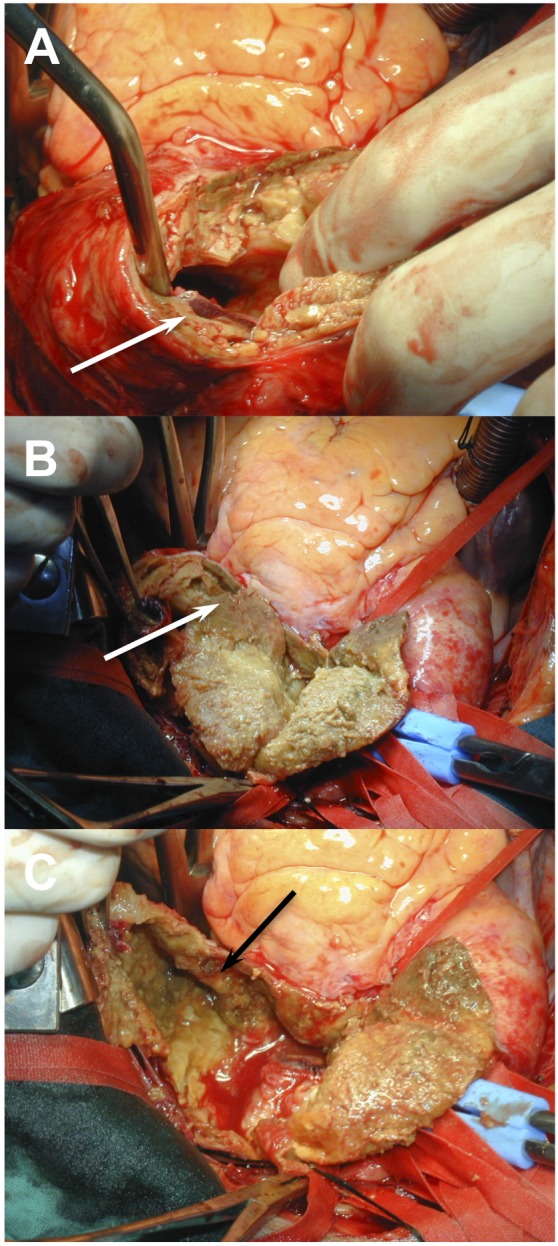
Important mural thrombus [3A and 3B (white arrow]. Thinned aneurysmal wall after thrombus removing [3C (black arrow)].

**Figure 4:**
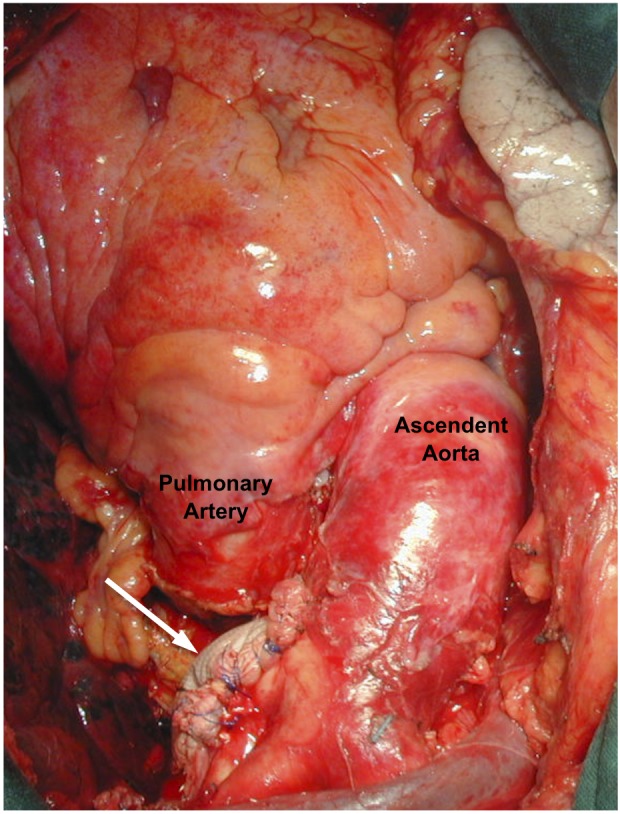
The aortic wall repair was made using a Dacron patch (white arrow) after the complete resection of the aneurysm.

In this case, we decided to treat the aortic pathology by open surgery instead of using a hybrid procedure [5] that comprises the endovascular graft exclusion of the aortic arch and the translocation of the supra-aortic arteries- due to the predominance of compressive symptoms and the important mural thrombus (Fig. 3). Thus, the patient was operated using partial cardiopulmonary byspass, without cardiac arrest, clamping the aneurismal neck. We proceeded to open and resect the aneurysm. Afterward we reconstructed the aortic wall with a Dacron patch and we reinforce the aortic wall with fibrin sealant (Fig. 4).

The patient course was favourable, improving his respiratory function, disappearing the dyspnea and dysphagia, and reducing the symptoms of hoarseness.

## Discussion

Hoarseness of voice, caused by a paralysis of the left recurrent laryngeal nerve due to left atrium enlargement in mitral valve stenosis, was described by Ortner in 1987. However, this syndrome, actually called Cardio-vocal syndrome, was associated with other specific cardiovascular pathologies, including severe pulmonary hypertension, congenital diseases, mitral valve disorders, iatrogenic causes or aortic aneurysm [4].

Hoarseness, in a cardio-vocal syndrome context, together with the presence of dysphagia, dyspnea and chest pain, constitutes a clear indication for the surgical treatment of aortic aneurysms [3]. The interventional technologies using hybrid procedures has decreased the mortality and morbidity for surgery of aortic arch aneurysms. We can distinguish two different types of hybrid procedures depending on the aortic arch approach [5]. Type I procedures, including the “frozen elephant trunk” are specially indicated in a concomitant aneurysms of aortic arch and descending aorta [6]. For its part, type II procedures consider the endovascular repair concept as the main technique, excluding the aortic arch with the stent graft. The open surgical step is limited to revascularize the supra-aortic arteries. Type II hybrid procedures is useful to treat lesions limited to the aortic arch in which the stent graft may exclude the arch successfully. Thus, in our case we could treat the aortic aneurysm using a type II hybrid procedure, preventing the aortic rupture with less risk for the patient. Nevertheless, probably we would not resolve the significant compressive symptoms with the isolated endovascular exclusion of the aneurysm, due to the giant size and the important mural thrombus.

In this case we decided to schedule the patient for open-surgery as the most appropriate surgical technique. With this approach we eliminated the morbidity from the compressive effect of adjacent structures, also preventing the aortic rupture. On the contrary, with the exclusion of the giant aneurysm by a stent graft we could not ensure the control of the compressive symptoms such as dysphagia, dyspnea or hoarseness.

We strongly recommend analyzing carefully the individual case and the clinical targets to resolve. In this sense, the new technologies are not always the most effective therapeutic response [7].
